# Dysphagia as a predictor of outcome and transition to palliative care among middle cerebral artery ischemic stroke patients

**DOI:** 10.1186/1472-684X-12-21

**Published:** 2013-05-10

**Authors:** Christa O’Hana V San Luis, Ilene Staff, Gilbert J Fortunato, Louise D McCullough

**Affiliations:** 1 Department of Neurology Hartford Hospital, Hartford, USA; 2 Departments of Neurology and Neurosciences The University of Connecticut Health Center, Farmington, USA; 3 The Stroke Center at Hartford Hospital, Hartford, USA; 4 Hartford Hospital Research Administration, Hartford, USA

## Abstract

**Background:**

Middle Cerebral Artery (MCA) territory strokes can be disabling and may leave patients unable to swallow safely. Decisions regarding artificial nutrition and goals of care often arise in patients with severe strokes leading to dysphagia. This study determined some predictors of early transition to palliative level of care among patients with acute ischemic MCA stroke with dysphagia.

**Methods:**

This is a retrospective cohort study. Demographic and clinical data of patients presenting to Hartford Hospital with an acute ischemic stroke between January 2005-December 2010 were gathered utilizing the Stroke Center at Hartford Hospital Database. The 236 patients included were divided into “early transition” and “not transitioned” to palliative care cohorts. Primary outcome was transition to palliative care. Factors that were significantly associated with an early transition to palliative level of care in univariate analysis were then entered into a multivariate logistic regression analysis to identify potential independent predictors of early transition to palliative level of care. The significance level was set at p < 0.05.

**Results:**

79 patients (34%) were transitioned to palliative level of care after failing the first swallow evaluation within a median of 3 days. Factors predictive of an early transition to palliative level of care after multivariate logistic regression analysis included advancing age (p < 0.001; OR: 1.10; 95% CI :1.056-1.155) , left MCA infarct (p = 0.039; OR: 0.417; 95% CI:0.182-0.956), a high NIHSS score on admission (p = 0.017; OR: 3.038; 95% CI: 1.22-7.555), administration of intra-arterial tPA (p < 0.001; OR: 7.106; 955 CI 2.541-19.873) and the inability to be assessed on the 1^st^ swallow evaluation (p < 0.001; OR 0.053; 95% CI 0.022-0.131).

**Conclusions:**

The severity of dysphagia influences early transition to palliative level of care in acute stroke patients. Independent predictors of an early transition to palliative level of care among patients with an acute MCA territory stroke and dysphagia included advancing age, a left MCA infarct, a high NIHSS score on admission, administration of intra-arterial tPA and the inability to be assessed on the 1^st^ swallow evaluation. This information may guide discussions with families of patients with MCA territory strokes regarding artificial nutrition and goals of care.

## Background

Approximately 795,000 people experience a first or recurrent stroke annually, 87% of which are ischemic in etiology [1]. 8%-12% of ischemic strokes result in death within 30 days. The mean age of patients experiencing early stroke associated mortality is 79.6 years old, leading to a 1-month case fatality rate of 8.1%. The most common location involved in ischemic stroke is the middle cerebral artery (MCA) territory [2]. It is well recognized that stroke leads to severe disability and functional dependence with a reported 5-year survival rate of 40% [3]. With these severe impairments, difficult decisions need to be made within several days of hospitalization regarding the aggressiveness of treatment including the use of life sustaining measures. Consideration of issues regarding goals of care and quality of life are crucial in this process. These decisions are especially pertinent in patients with severe strokes that leave the patient unable to swallow safely or protect his or her airway.

The role played by palliative care is very evident in these cases and the need for family support and the clear establishments of goals of care are critical [4,5]. Palliative care, according to the World Health Organization (WHO) is “an approach that improves the quality of life of patients and their families facing the problems associated with life-threatening illness, through the prevention and relief of suffering by means of early identification and impeccable assessment and treatment of pain and other problems, physical, psychosocial and spiritual” [6]. The proportion of patients referred to palliative care with a diagnosis of stroke ranges from 6.5%-73.8% depending on the study examined [7-9]. In one study, 10% of palliative care consults centered around issues directly related to artificial hydration or feeding [9]. Possible disagreement between families regarding fluids and feeding was found in 46% of family interactions [10]. It is a challenge to determine the best timing for palliative care consults in the setting of acute stroke. We hypothesized that dysphagia in middle cerebral artery (MCA) stroke patients, based on failure in a formal swallow evaluation or the inability to be assessed for a swallow study due to poor neurological status would be a significant determinant of an early transition to a palliative level of care. There are no studies available examining predictors of early transition to palliative level of care and the influence of dysphagia in palliative care decision making in this cohort of patients. This study determined the predictors of early transition to palliative level of care among patients with acute ischemic stroke in the MCA distribution in the setting of dysphagia. This information may be useful for the early establishment of goals of care and assist in decision making in patients with acute ischemic stroke.

## Methods

### Study design

This study is a retrospective analysis of existing data in the Stroke Center at Hartford Hospital Database. Data from patients presenting to Hartford Hospital with an acute ischemic stroke between January 2005 and December 2010 were retrospectively reviewed. This study was reviewed and approved by the Hartford Hospital IRB. The Stroke Center at Hartford Hospital (SCHH) is certified by the Joint Commission of Accreditation for Healthcare Organizations (JCAHO) as a Primary Stroke Center and serves as a tertiary referral center.

### Participants

**Figure 1:**
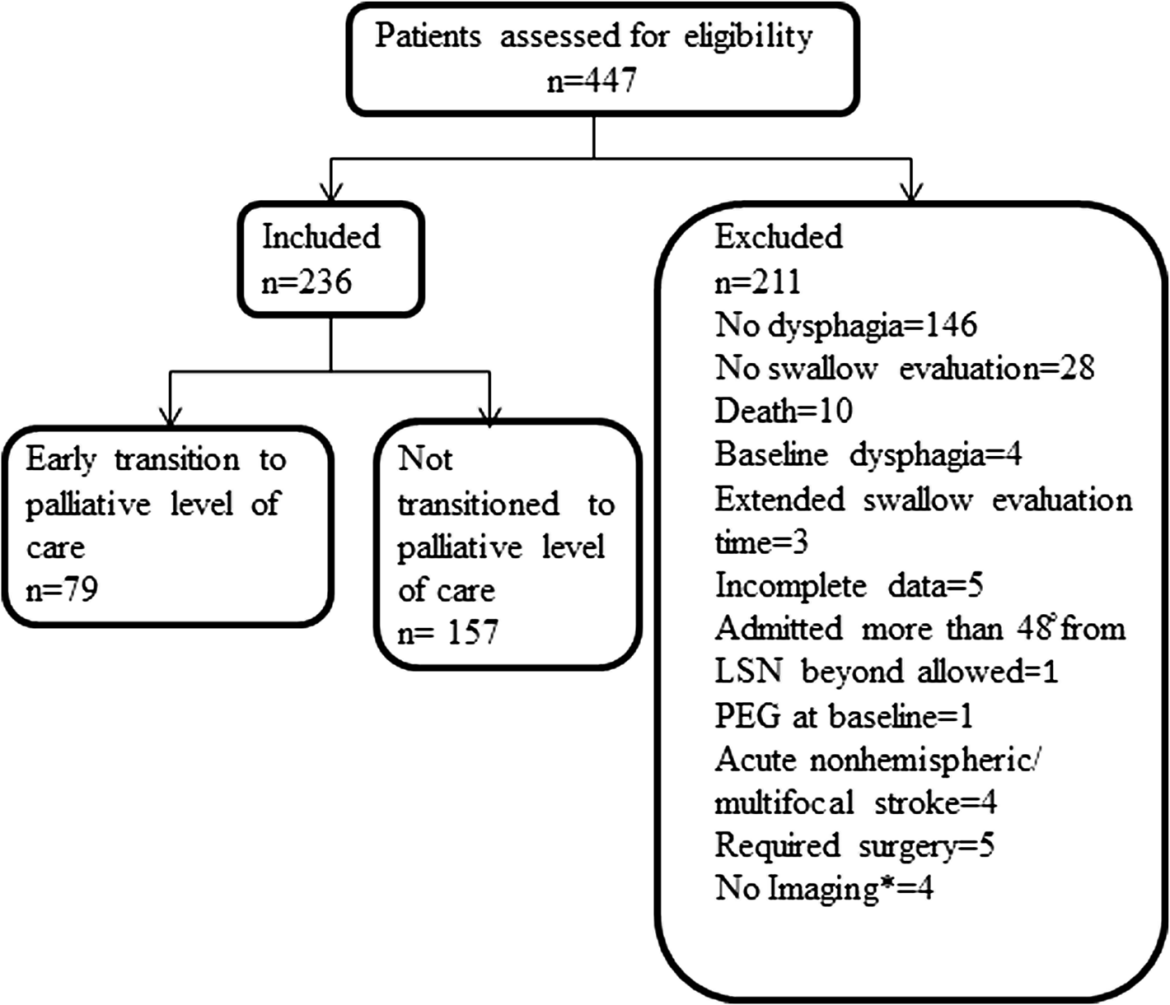
**Subject selection.** LSN = last seen normal, PEG = percutaneous endoscopic gastrostomy.

A total of 447 patients identified from the Hartford Hospital Stroke Center database to have either a left or right MCA distribution acute ischemic stroke (AIS) admitted in the period of January 2005- December 2010 were initially screened for inclusion in this study. Patients were excluded if there was no evidence of stroke on brain magnetic resonance imaging (MRI) or Computed Tomography (CT) scan performed within 48 hours of admission, if they were admitted beyond 12 hours (or were transferred from an outside facility) or were ≥ 48 hours (for a patient from home or facility) from symptom onset or time “last seen normal” (LSN). Patients were also excluded if they had no dysphagia as evaluated by speech therapy utilizing the Hartford Hospital Dysphagia Rating Scale (see Additional file 1) which was based on the Dysphagia Outcome and Severity Scale [11], or if there was preexisting dysphagia (noted by speech therapy) due to other comorbid conditions that are known to affect swallowing (including head and neck cancers, neurodegenerative disorders, neuromuscular disorders and connective tissue disease). Patients who underwent surgery for hemispheric strokes with significant mass effect, those with acute nonhemispheric or multifocal strokes, patients whose swallow evaluation was delayed beyond 24 hours from admission or beyond 48 hours from admission (if a patient went to procedures when the swallow evaluation was to be conducted) were also excluded. Those with percutaneous endoscopic gastrostomy (PEG) tube at baseline were also excluded from subsequent analysis. Two hundred thirty six patients were included in the study (Figure 1). The patients were divided into the “Early transition” and “Not transitioned” to palliative care cohort. Early transition patients are those who were seen by a speech therapist post stroke that were unable to undergo the swallow evaluation or failed the first swallow evaluation, which were then transitioned to palliative level of care. All patients were initially offered artificial feeding if unable to swallow whether or not they were transitioned early to palliative care. The decision to transition to palliative level of care was made by a legally designated patient representative after a family meeting with the neurology attending and palliative care representative (either a palliative care physician or trained advanced practice nurse, registered nurse or social worker). These meetings include a discussion of imaging findings, swallow evaluation result, probable functional outcome of the patient, goals of care and artificial nutrition. Data regarding the specifics of the meeting and discussion leading to transition to palliative care were not available as often the content of each individual meeting was not described in detail. No standardized protocols currently exist for transition to palliative care.

### Description of the institution stroke coverage

Hartford hospital is covered by one “Stroke/ Neurovascular” attending from Monday to Friday. The attending changes each week. The weekend coverage begins at 1:00 p.m. on Fridays and ends on Mondays 8:00 a.m. During the weekend, the covering attending, who may not necessarily be a “Stroke/ Neurovascular” attending oversees all the stroke and non-stroke patients in the neurology in-patient service. Family meetings held during the weekdays are held in the presence of the stroke attending, with or without the palliative care service representative who may be a palliative care medicine physician, advance practitioner nurse, registered nurse or social worker. All family members who are critical to the decision making and who can be available for the meetings are included. Findings of the swallow evaluations, functional prognosis and goals of care issues are discussed by the stroke attending. If a palliative care representative is present, goals of care may be discussed by them. During the weekends, goals of care discussion may be done by the covering attending who may not necessarily be a stroke attending. The palliative care representative was not available to attend family meetings on weekends but was accessible by phone if needed. At times, if the covering attending is a non-stroke attending, goals of care discussions are postponed for the weekday.

### Data collection

The Hartford Hospital Stroke Center database was examined to gather demographic (e.g. age, race, gender, comorbidities) and clinical (e.g. day of the week the patient was admitted, location of stroke, NIHSS on admission, swallow evaluation, intervention done) data of patients with MCA stroke admitted during the stated period. The paper charts of the corresponding patients were reviewed to determine additional clinical data regarding palliative level of care status relative to swallow evaluation.

### Statistics

Descriptive statistics were compiled for all factors and outcome variables. Proportions were used for dichotomous or other categorical variables. Means and standard deviations or medians and interquartile ranges were calculated for continuous variables as appropriate for their underlying distributions (approximating normal or not). The primary outcome was early transition to palliative level of care in patients identified with dysphagia. Patients who were transitioned to palliative care were compared to others on demographics (age, race, gender), comorbidities, admission National Institute of Health Stroke Scale (NIHSS) score [12], treatment received (intraarterial [IA] and intravenous [IV] tissue plasminogen activator [tPA] administration, interventional procedure done) and stroke laterality (right versus left). Dichotomous or categorical variables were analyzed with chi-square test of proportions. Continuous variables (e.g. age) were compared for mean differences using a t-test for independent groups. The NIHSS which is a continuous scale but not normally distributed was analyzed with the Wilcoxon Rank Sum Test. In additional analysis, age was dichotomized at 70 years and the NIHSS was separated into three categories (0–6, 7–15 and 16+) based on increasing stroke severity. Factors showing a significant univariate relationship with the transition to palliative care were then entered into a multivariate logistic regression analysis to identify potential independent predictors of early transition to palliative level of care. The significance level was set at p < 0.05 for all analyses; data were extracted from an ACCESS database into Excel where data were confirmed and expanded. All data were imported into Statistical Package for the Social Sciences (SPSS) v.14 for analyses.

## Results

A total of 236 patients were deemed eligible for the study. The patients were divided into “early transition” and “not transitioned” cohorts. 79 (34%) patients who transitioned to palliative care failed the first swallow evaluation or were unable to be formally assessed for dysphagia by speech therapy due to a decreased level of consciousness.

Table 1 compares the baseline characteristics of patients transitioned to palliative level of care versus those who were not. The mean age of the study population is 78 ± 14. Most are white females and majority had hypertension. Patients who were transitioned to palliative level of care were significantly older (83 years old vs. 76 years old, p = 0.003), with higher rates of atrial fibrillation (59% vs. 41%, p = 0.011). The median time from admission to the transition to palliative level of care (data available in 48 patients of this population) was 3 (IQR 2,5) days. The majority of those patients who had been able to undergo the first swallow evaluation in the group who were transitioned to palliative care had moderate to severe dysphagia (86% vs. 24%, p = <0.001) while the majority of those who had not been transitioned had mild-moderate dysphagia (76% vs. 14%, p = <.001).

**Table 1:** Baseline characteristics of patients transitioned to palliative level of care versus patients not transitioned to palliative level of care

**Variable**	**All patients (n = 236)**	**Patients transitioned to palliative level of care (n = 79)**	**Not transitioned to palliative level of care (n = 157)**	**p-value**
Age	236			**.003**
<70 n (%)	57 (24%)	10 (13%)	47 (30%)	**.003**
>70 n (%)	179 (76%)	69 (87%)	110 (70%)	
Mean (SD)	77.8 (13.58)	82.72 (10.61)	75.17 (14.22)	
Race	232			.207
White n (%)	208 (90%)	70 (89%)	138 (91%)	
Black n (%)	10 (4%)	2 (3%)	8 (5%)	
Hispanic n (%)	13 (6%)	7 (9%)	6 (4%)	
Gender	237			.802
Female n (%)	149 (63%)	49 (62%)	100 (64%)	
Male n (%)	87 (37%)	30 (38%)	57 (36%)	
AF* n (%)	110 (47%)	46 (58%)	64 (42%)	**.011**
HTN* n (%)	195 (83%)	63 (80%%)	132 (84%)	.407
Dementia n (%)	32 (14%)	12 (15%)	20 (13%)	.604
Dysphagia severity on 1^st^ swallow evaluation				**<.001**
Mild to Moderate	94 (70%)	2 (14%)	92 (76%)	
Moderate to severe	41 (30%)	12 (86%)	29 (24%)	

*AF-atrial fibrillation, HTN-hypertension.

Table 2 demonstrates the findings from univariate analysis in the patients who were transitioned to palliative level of care versus those that were not. Factors associated with an early transition to palliative level of care in the setting of dysphagia included left MCA territory stroke (70% vs. 47%, p = 0.001), higher median NIHSS on admission (19 [IQR 16,23] vs. 15 [IQR 8,19], p < 0.001), intra-arterial tPA use (38% vs. 9%, p <0.001), atrial fibrillation (58% vs. 42%), weekday admission (80% vs. 59%, p = 0.001) and inability to be assessed on 1^st^ swallow evaluation (82% vs. 23%, p = <.001). 36 (36%) of the patients who were not able to undergo the 1^st^ swallow evaluation did not transition to palliative level of care. 12 (33%) of these patients subsequently underwent PEG tube placement.

**Table 2:** Univariate analysis of possible predictors of early transition to palliative care

**Variable**	**All patients (n = 237)**	**Patients transitioned to palliative level of care (n = 80)**	**Patients not transitioned to palliative level of care (n = 157)**	**p- value**
Location				**.001**
Left MCA n (%)	129 (55%)	55 (70%)	74 (47%)	
Right MCA n (%)	107 (45%)	24 (30%)	83 (53%)	
Admit NIHSS score				**<.001**
				**<.001**
0–6 n (%)	28 (12%)	4 (5%)	24 (15%)	
7–15 n (%)	69 (30%)	14 (18%)	55 (35%)	
16 and higher n (%)	136 (58%)	58 (76%)	78 (50%)	
Median (IQR*)	17 (10,20)	19 (16, 23)	15 (8,19)	
Intraarterial tPA n (%)	44(19%)	30 (38%)	14 (9%)	**<.001**
Intravenous tPA n (%)	89 (38%)	30 (38%)	59 (38%)	.953
Use of Device (n = 26)	26 (11%)	7 (9%)	19 (12%)	.453
Day of Week				**.001**
Weekend n (%)	81 (34%)	16 (20%)	65 (41%)	
Weekday n (%))	155 (66%)	63 (80%)	92 (59%)	
Ability to be assessed on 1^st^ swallow evaluation				**<.001**
Can n (%)	135 (57%)	14 (18%)	121 (77%)	
Cannot n (%)	101 (43%)	65 (82%)	36 (23%)	

*MCA- middle cerebral artery, NIHSS – national institutes of health stroke scale, IQR = interquartile range, tPA- tissue plasminogen activator.

In multivariate logistic regression analysis (Table 3), the same variables were independent predictors of early transition to palliative level of care in the setting of dysphagia in patients with MCA AIS except for admission day (weekday vs. weekend).

**Table 3:** Multivariate logistic regression analysis of statistically significant predictors of early transition to palliative level of care

**Variable**	**Sig**	OR* (95% CI)
Age	**<0.001**	**1.105 (1.056-1.155)**
Atrial fibrillation	0.529	0.754 (0.313-1.816)
Left vs Right Location of infarct	0.**039**	**0.417 (0.182-0.956)**
Admit NIHSS score*	**0.017**	**3.038 (1.222-7.555)**
Intraarterial tPA	**<0.001**	**7.106 (2.541-19.873)**
Weekday vs. weekend patient admission	0.239	1.690 (0.706-4.049)
Ability to be assessed on 1^st^ swallow evaluation	**<0.001**	**0.053 (0.022-0.131)**

*NIHSS-national institutes of health stroke scale score, tPA- tissue plasminogen activator, OR- odds ratio.

## Discussion

This study shows that dysphagia factors, specifically dysphagia severity and the inability to be assessed on the first swallow evaluation due to lethargy, influence early transition to palliative level of care among patients with acute MCA territory stroke. Independent predictors of early transition to palliative level of care in the setting of an acute MCA territory stroke in patients with dysphagia included advancing age, a left MCA infarct, a high NIHSS on admission, administration of intra-arterial tPA and the inability to be assessed on the 1^st^ swallow evaluation.

Literature on the role of palliative management of acute stroke patients and the timing of palliative care consult are sparse [13]. In the acute phase of stroke, it is challenging to predict functional outcome. The decision to withdraw care is usually based on prognosis and functional outcome, associated co-morbidities, available treatment options and family and/or patient wishes and values [14]. There are many cases where continuing life-sustaining interventions may be considered to be an inappropriate overuse of medical resources; however, aggressive interventions may be needed to give patients time to recover, especially in patients receiving thrombolytics. Clearly, ability to swallow is a major factor in this decision making. Patients that either fail an initial swallow evaluation, or are too obtunded to perform this evaluation, are more likely to transition to palliative care. In addition, patients who were able to undergo the swallow evaluation that were subsequently transitioned to palliative care were noted to have moderate to severe dysphagia. A physician’s prognosis significantly affects these decisions [14] and because of that, any information that is available may be helpful in discussions with families regarding care. Most of the time, family conversations regarding administration of artificial nutrition (through a dobhoff tube, nasogastric tube or percutaneous gastrostomy tube placement) are often a critical decision making period for patients' families. Holloway et al. (2010) have noted that the most common issue discussed when a palliative care consult is initiated involves artificial nutrition/ feeding tubes [15].

In this study 34% of patients with MCA AIS are transitioned to palliative level of care during their initial hospitalization. This is considerably lower than what was previously reported by Blaquire and colleagues [10] however in that study patients with hemorrhagic strokes who have poorer prognosis [16] were also included. The number of patients transitioned to palliative care is still quite high, and likely reflects the severe disability and poor functional outcomes that are associated with large cortical strokes. The patients were transitioned within a median of 3 days of admission, a time frame similar to what has been reported in the literature [10] although, almost half (48%) of patients were transitioned on day 0. Further studies analyzing clinical implications of “time to early transition to palliative care” and the effect on families, healthcare costs and administration are needed.

Consistent with previous literature [9,15], we found that age is a major factor in treatment decisions. Older patients were significantly more likely to transition to early palliative care. Increasing co-morbid medical illnesses may be a major factor although this was not seen in the present study. In this study, a higher NIHSS score on admission was a significant predictor of the transition to palliative care. In a recent review by Mazzocato of patients referred to palliative care, the median NIHSS was 21 [9], quite similar to what was found in this study (median of 19 [IQR 16,23]). A higher NIHSS is usually indicative of an impairment of language, as the NIHSS is heavily weighted to add points for aphasia. Indeed, patients with right hemispheric infarcts with high NIHSS have larger infarct volumes compared to left hemispheric stroke patients with the same NIHSS due to this bias [17]. Severe aphasia is a disabling and isolating deficit, and indeed in this study, patients were more likely to transition to palliative care if they had left hemispheric infarcts. Certainly, aphasia leads to a higher NIHSS but could also lead the physician to predict a poorer functional outcome and quality of life during family meetings.

Interestingly, administration of intra-arterial tPA was also associated with the transition to palliative care. As only patients with the most severe strokes (often carotid or trunk MCA occlusions) with clear large vessel occlusion noted on imaging are candidates for interventional therapies, if re-canalization does not occur, subsequent infarcts are very large. This is associated with extremely poor functional outcomes. The association of transition to palliative care with weekday admission may simply reflect the availability of the palliative care team on weekdays. These results must be interpreted with caution, as this study has selected for a very small subset of patients, making bias more likely. In addition, not every weekend is covered by a stroke specialist, as approximately half of the weekend coverage is provided by non-stroke neurologists. It is possible that stroke neurologists may be more comfortable in prognosticating the poor outcomes expected from large middle cerebral artery infarcts.

This study has several limitations which must be recognized when interpreting our findings. Most importantly, this is a retrospective analysis of patient data from a single institution. Patients were pre-selected on the location of their stroke (MCA) and their ability to undergo an initial swallow evaluation; therefore these results cannot be generalized to other patient populations. Furthermore, the relatively small number of subjects limits its generalizability to other stroke patient populations. Data regarding the specific content of family meetings prior to transitioning to palliative care are not available. Despite the mentioned limitations, the information in this study could be used as discussion points when describing certain characteristics in MCA territory stroke patients that predict poor outcome. Future prospective studies on outcomes, possibility of swallow re-evaluation, effect on family and patient decision making, and application to healthcare costs are encouraged.

## Conclusions

Although this study is limited, we have shown that in a cohort of MCA stroke patients, dysphagia severity influences early transition to palliative level of care. In this cohort, independent predictors of early transition to palliative level of care include age greater than 70, left MCA territory strokes, higher admission NIHSS score, intra-arterial tPA use and the inability to be assessed on the initial swallow evaluation. This knowledge may aid families in early establishment of goals of care and in decision making in patients with acute ischemic stroke.

## Competing interests

The authors declare that they have no competing interests.

## Authors’ contributions

CSL conceived of the study, participated in the design and coordination, and drafted the manuscript. LM participated in the design and coordination, drafted and edited the manuscript. IS participated in the design and coordination, drafted and edited the manuscript and performed the statistical analysis. GF participated in the design and coordination and drafted and edited the manuscript. All authors read and approved the final manuscript.

## Pre-publication history

The pre-publication history for this paper can be accessed here:

http://www.biomedcentral.com/1472-684X/12/21/prepub

## Supplementary Material

Additional file 1 **Appendix 1.** Hartford hospital dysphagia rating scale. Click here for file
