# Supplementary material for: Dysphagia as a predictor of outcome and transition to palliative care among middle cerebral artery ischemic stroke patients
Source: BMC Palliat Care. 2013 May 10;12:21. doi: 10.1186/1472-684X-12-21 (PMC3665461; doi:10.1186/1472-684X-12-21)
Supplement: Additional file 1 — Appendix 1. Hartford hospital dysphagia rating scale. [file 1472-684X-12-21-S1.pdf]

HARTFORD HOSPITAL DYSPHAGIA RATING SCALE (DRS)

FULL P.O. NORMAL DIET

Level 7: Normal in all situations

- Normal Diet
- No strategies or extra time needed

Level 6: Within Functional Limits/ Modified Independence

- Normal Diet, Functional swallow
- Patient may have mild oral or pharyngeal delay, retention or trace epiglottal undercoating BUT
- Independently and spontaneously compensates/ clears
- May need extra time meal
- NO aspiration or penetration across consistencies

FULL P.O.: MODIFIED DIET AND/ OR INDEPENDENCE

Level 5: Mild Dysphagia: distant Supervision needed, May need one diet consistency restricted (Functional after set-up and/ or initial instruction)

MAY EXHIBIT 1 OR MORE OF THE FOLLOWING

- Aspiration of thin liquids only but with strong reflexive cough to clear completely
- Airway penetration midway to cords with 1+ OR to cords with one consistency but *clears spontaneously*
- Retention in pharynx which is *cleared spontaneously*
- Mild Oral Dysphagia with reduced mastication +/ or oral retention which is *cleared spontaneously*

Level 4: Mild- Moderate Dysphagia: Intermittent Supervision/ Cuing Needed. 1-2 consistencies restricted

MAY EXHIBIT 1 OR MORE OF THE FOLLOWING:

- Retention in pharynx *cleared with cue*
- Retention in the oral cavity which is cleared with cue
- Aspiration with one consistency, *with* weak or no reflexive cough
- OR Airway penetration to the level of the vocal cords *with* cough with 2 consistencies
- OR Airway penetration to the level of the vocal cords *without* cough with 1 consistency

Level 3: Moderate Dysphagia: Total Assist, Supervision or Strategies Needed 2+ diet consistencies restricted (**CAN SAFELY TOLERATE AT LEAST 2 CONSISTENCIES**)

MAY EXHIBIT 1 OR MORE OF THE FOLLOWING:

- Moderate retention in pharynx, cleared *with* cue
- Moderate retention in oral cavity, cleared *with* cue
- Airway penetration to the level of the vocal cords *without* cough with 2 or more consistencies
  OR Aspiration with 2 consistencies, with *weak or no reflexive cough*
  OR Aspiration with 1 consistency, *no cough* AND Airway penetration to cords with 1, no cough

NON-ORAL NUTRITION NECESSARY

Level 2: Moderately Severe Dysphagia: Maximum Assistance or Max Use of Strategies with Partial P.O. only: (**TOLERATES AAT LEAST 1 CONSISTENCY SAFELY WITH TOTAL USE OF STRATEGIES**)

MAY EXHIBIT 1 OR MORE OF THE FOLLOWING:

- Severe retention in pharynx, unable to *clear or needs multiple cues*
- Severe oral stage bolus loss or retention, *unable to clear or needs multiple cues*
- Aspiration with 2 or more consistencies, *no reflexive cough, weak volitional cough*
  OR Aspiration with 1 + consistency, no cough AND Airway penetration to cords with 1+, *no cough*

Level 1: Severe: NPO, Unable to tolerate any p.o. safely:

- Severe retention in pharynx, *unable to clear*
- Severe oral stage bolus loss or retention, *unable to clear*
- Silent aspiration with 2 or more consistencies, nonfunctional volitional cough
- Or unable to achieve swallow
